# Genetic factors influencing frontostriatal dysfunction and the development of dementia in Parkinson's disease

**DOI:** 10.1371/journal.pone.0175560

**Published:** 2017-04-11

**Authors:** Ismael Huertas, Silvia Jesús, Francisco Javier García-Gómez, José Antonio Lojo, Inmaculada Bernal-Bernal, Marta Bonilla-Toribio, Juan Francisco Martín-Rodriguez, David García-Solís, Pilar Gómez-Garre, Pablo Mir

**Affiliations:** 1 Unidad de Trastornos del Movimiento, Servicio de Neurología y Neurofisiología Clínica, Instituto de Biomedicina de Sevilla (IBiS), Hospital Universitario Virgen del Rocío/CSIC/Universidad de Sevilla, Seville, Spain; 2 Servicio de Medicina Nuclear. UDIM. Hospital Universitario Virgen del Rocío, Seville, Spain; 3 Centro de Investigación Biomédica en Red sobre Enfermedades Neurodegenerativas (CIBERNED), Madrid, Spain; Oslo Universitetssykehus, NORWAY

## Abstract

The *dual syndrome* hypothesis for cognitive impairment in Parkinson's disease (PD) establishes a dichotomy between a frontrostriatal dopamine-mediated syndrome, which leads to executive deficits, and a posterior cortical syndrome, which leads to dementia. Certain genes have been linked to these syndromes although the exact contribution is still controversial. The study’s objective was to investigate the role of *APOE*, *MAPT*, *COMT*, *SNCA* and *GBA* genes in the dual syndromes. We genotyped *APOE* (rs429358 and rs7412), *MAPT* (rs9468), *COMT* (rs4680) and *SNCA* (rs356219) risk polymorphisms and sequenced *GBA* in a cohort of 298 PD patients. The degree of dopaminergic depletion was investigated with [^123^I]FP-CIT SPECTs and the presence of dementia was ascertained with a long-term review based on established criteria. The association between genetic and imaging parameters was studied with linear regression, and the relationship with dementia onset with Cox regression. We found that *APOE2* allele (*P*_*put*_ = 0.002; *P*_*cau*_ = 0.01), the minor allele 'G' in *SNCA* polymorphism (*P*_*put*_ = 0.02; *P*_*cau*_ = 0.006) and *GBA* deleterious variants in (*P*_*put*_ = 0.01; *P*_*cau*_ = 0.001) had a detrimental effect on striatal [^123^I]FP-CIT uptake in PD. Conversely, Met/Met carriers in *COMT* polymorphism had increased caudate uptake (*P*_*cau*_ = 0.03). The development of dementia was influenced by *APOE4* allele (HR = 1.90; *P* = 0.03) and *GBA* deleterious variants (HR = 2.44; *P* = 0.01). Finally, we observed no role of *MAPT* locus in any of the syndromes. As a conclusion, *APOE2*, *SNCA*, *COMT* and *GBA* influence frontostriatal dysfunction whereas *APOE4* and *GBA* influence the development of dementia, suggesting a double-edged role of *GBA*. The dichotomy of the dual syndromes may be driven by a broad dichotomy in these genetic factors.

## Introduction

Cognitive impairment is a common and disabling non-motor symptom of Parkinson's disease (PD). Cognitive deficits may be present in up to 24% of PD patients by the time of diagnosis, and this rate reaches over 80% in the long-term [1]. Although dysexecutive syndrome has long been considered the main hallmark of cognitive decline in PD, deficits in visuospatial, memory and attention functions may be also present. The rate and pattern of these deficits vary greatly among PD patients, and different biological mechanisms appear to play a role [2]. In this regard, the *dual syndrome* hypothesis was recently proposed, suggesting two facets of cognitive decline in PD: (i) changes in frontostriatal dopaminergic transmission, leading to deficits in planning, working memory, response inhibition and attentional control; and (ii) posterior cortical Lewy body pathology and secondary cholinergic loss, affecting visuospatial, mnemonic and semantic functions and leading to dementia [3].

It is possible to assess the state of the frontostriatal circuitry by imaging the striatal dopamine transporter (DAT) with [^123^I]FP-CIT SPECT. In this scan, dopamine depletion can first be observed in the putamen, which affects the motor loop, whilst dopamine depletion in the caudate usually occurs in later stages, affecting two well-defined frontostriatal loops: the cognitive and the limbic loops [4]. Therefore, the integrity of this latter pathway is essential to correct cognitive functioning, and a large number of studies have in fact found a correlation between cognitive performance (including executive and working memory tasks) and caudate dopamine levels in PD [5].

Several genetic *loci* have also been proposed as risk factors for cognitive decline in PD [6]. Some of these genetic *loci* have been linked to the dopaminergic pathway, such as the Val158Met polymorphism in the catechol-O-methyltransferase gene (*COMT*). This gene encodes the COMT enzyme, which contributes to the degradation of cortical dopamine. Met carriers show low enzyme activity in comparison to Val carriers. This genotype therefore modulates dopamine levels in the frontostriatal network, and in turn, executive function performance [7].

Other genetic *loci* have been linked to both the development of dementia and performance in tasks mediated by other non-dopaminergic mechanisms. Specifically, the apolipoprotein E gene (*APOE*) ε4 allele has been associated with an increased risk of dementia [8] and deficits in memory and verbal fluency [7, 9]. Furthermore, the microtubule-associated protein tau gene (*MAPT*) H1 haplotype has been linked with dementia [10, 11] and visuospatial deficits [7], although recent results have been controversial [9]. It is not yet known what role these two *loci* play in dopaminergic degeneration, and this needs to be addressed.

Another genetic *locus* of interest is the rs356219 polymorphism, located in the 3’UTR of the α-synuclein gene (*SNCA*). Mutations and repetitions in *SNCA* lead to a familial form of PD with prominent cognitive impairment and dementia. The rs356219 polymorphism has been linked to PD pathogenesis [12], although its role in cognition is far from clear. Its relationship with dopaminergic imaging has besides not yet been studied. Lastly, the glucocerebrosidase gene (*GBA*) is the most common genetic factor that has yet been identified for developing PD [13]. PD patients with mutations in *GBA* have earlier disease onset, and are at a higher risk of developing visual hallucinations, cognitive impairment and dementia [14]. Recent studies suggest that *GBA*-carriers have a more severe phenotype, with quicker disease progression [15]. As is the case with other genes, very little is known about the relationship between *GBA* and dopaminergic imaging.

Although other groups are thoroughly investigating the relationship between these genes and domain-specific neuropsychological tasks, using large cohorts of PD patients, not enough studies have yet been conducted to evaluate how these genetic loci contribute to dopaminergic degeneration through imaging. Furthermore, recent studies have produced contradictory results concerning the role that some *loci*, such as the H1 haplotype in *MAPT*, play in PD dementia. This study aims to investigate the role that these genes play in striatal dopaminergic denervation and PD dementia. To this end, we collected [^123^I]FP-CIT SPECT images and long-term clinical data on the developement of dementia, and genotyped *APOE*, *MAPT*, *COMT* and *SNCA* risk polymorphisms, as well as *GBA* screening in a cohort of 298 PD patients from our centre.

## Materials and methods

### Subjects

A total of 298 PD patients were included in this study (age at onset 55 ± 13 years, 60% males), recruited from the Movement Disorders Unit at Virgen del Rocío Hospital (Seville, Spain). The diagnosis of PD was made using the UK Parkinson's Disease Society Brain Bank clinical diagnostic criteria. All patients underwent [^123^I]FP-CIT SPECT (mean disease duration 6 ± 6 years, median Hoehn and Yahr 2 [1.5, 2.5]) and were clinically monitored during the course of the disease with periodic visits to our clinic. At SPECT exam, 17% of the patients had no medication, 17% on levodopa, 18% on dopaminergic agonists and 48% on both. The median levodopa equivalent daily dose (LEDD) for those under treatment was of 596 [300, 1063] mg/day. The influence of the genetic factors on the dopamine-mediated syndrome was investigated through the association between the genotypes and striatal DAT, whereas the influence on the posterior syndrome was investigated through the association between the genotypes and the onset of dementia. We identified patients who met diagnostic criteria for possible or probable dementia [16] in a long-term review of the medical records (mean disease duration at time of review: 11 years). The diagnosis of dementia was ascertained by a variety of screening tools including a medical interview to the patient and caregiver, or scores in standard scales such as Mini Mental State Examination (MMSE ≤ 24), and Parkinson’s disease Dementia Short Screen (PDD-SS ≤ 11) [17]. The disease duration at the visit when the patient met the criteria was used to perform survival analyses of the progression to dementia. The core features of these criteria include the presence of deficits (social, occupational, or personal care) impairing daily life and the presence of deficits in one or more cognitive domains such as attention, executive, visuo-spatial, memory and language, and behavioral symptoms. The diagnosis of possible dementia included an atypical profile of cognitive impairment in one or more domains such as prominent or receptive-type (fluent) aphasia, or pure storage-failure type amnesia. The diagnosis of probable dementia included impairment in at least two domains such as attention, executive and/or visuo-spatial functions, and free recall memory. Probable dementia diagnosis was also reinforced by the presence of behavioral symptoms such as apathy, changes in personality and mood, hallucinations and delusions, and excessive daytime sleepiness. All subjects provided informed written consent, and the Hospital Virgen del Rocío ethics committee approved this study.

### Genetics

Genomic DNA was extracted from peripheral blood samples using the standard methods. All patients underwent genotyping for rs429358 and rs7412 (*APOE* ε2, ε3, and ε4), rs9468 (*MAPT* H1 vs. H2), rs4680 (*COMT* Val158Met), and rs356219 (*SNCA*). Genotyping was performed with TaqMan SNP Genotyping Assay in a LightCycler480 (Roche Applied Science), and the genotyping success rate was over 98%. All patients were also screened for variants in the entire gene *GBA*, using both high-resolution melting analysis for all exons and direct DNA resequencing for those samples showing abnormal melting profiles. Of the 298 subjects, we identified 62 *GBA* variants in a total of 48 carriers. Identified variants were classified as potential deleterious (n = 35) or potential benign (n = 27) (see S1 Table) based on *in silico* analyses using the bioinformatic tools Grantham score, Polyphen-2, MutPred v1.2, and Mutation Taster. PD patients were classified into the group of “benign” if all the carried variants were potentially benign (n = 17), and classified into the group of "deleterious" if at least one of the carried variants was potentially deleterious (n = 31). A more detailed description about the sequencing procedure, variants and criteria for assessing pathogenicity can be found in a recent work from our group [18].

### SPECT imaging

The acquisition procedure and SPECT reconstruction can be found in a previous report [19]. SPECT images were processed with standard procedures in SPM8 using a homemade [^123^I]FP-CIT template (http://www.nitrc.org/projects/spmtemplates). Quantitative analyses were based on volumes of interest in the striatum manually drawn by expert nuclear-medicine specialists (https://www.nitrc.org/projects/striatalvoimap) following established methodology [20]. A volume in the occipital cortex was used as a reference region and, for each patient, [^123^I]FP-CIT binding potential (BP) for posterior putamen and head of caudate was calculated. The BP was expressed as the percentage of age-expected binding with respect to 184 normal scans (age range 18–90 years) [21], and since laterality can affect the statistics at the group level, the comparisons were made for the more affected side.

### Statistical analysis

We investigated the role of *APOE* ε2 and ε4 alleles; *MAPT* H1 and H2 haplotypes; *COMT* Met allele; *SNCA* G allele; and deleterious and benign variants in *GBA*. Based on the previous reported genotypes of risk in the literature, the comparisons of interest in this study were: *APOE*: ε2+ vs. (ε2-, ε4-) and ε4+ vs (ε2-, ε4-); *MAPT*: H1/H1 vs. H2; *COMT*: Met/Met vs. Val; *SNCA*: G vs. A; *GBA*: GBA+ vs. non carriers. Other potential genetic models were also explored (allelic, dominant and recessive), but for the sake of simplicity only the contrasts of interest and/or the strongest associations are presented. We performed separate linear regression analyses for each gene and imaging variable to study their potential interaction with PLINK (http://pngu.mgh.harvard.edu/~purcell/plink/). We entered the quantitative imaging variables as dependent variables, the genetic factors as independent variables and the regression coefficients were calculated as a measure of effect size. The potential confounding factors sex, age and disease duration at time of scan were also introduced as covariates. Although previous studies have shown that dopaminergic medications do not alter DAT imaging [22, 23], we verified with exploratory analyses that medication was not confounding striatal uptake. The development of dementia was examined with survival analyses through Cox regression. For this analysis, we used as event variable the presence of dementia (yes/no), and as time variable the disease duration at dementia onset for the positive cases (yes) and the disease duration at the review of the records for the negative cases (no). We performed a separate regression analysis for each gene and Hazard ratios (HR) for each risk genotype were calculated adjusting for sex and age at disease onset as potential confounding factors for dementia. Analyses were done using IBM SPSS Statistics 22.0 and the statistical threshold for significance was set to *P* < 0.05. Given that this is an exploratory study, we did not apply multiple testing penalization.

## Results

### Dopaminergic imaging

Distribution of genotypes and descriptive values for putamen and caudate age-expected [^123^I]FP-CIT BP are shown in Table 1. Age at onset was similar among genotypes for each gene except for *GBA*, for which carriers of variants were younger than non-carriers (51 vs. 55 years; *P* = 0.004). In linear regression analyses, we found that *APOE* ε2 allele, the minor allele 'G' in *SNCA* polymorphism, and deleterious variants in *GBA* were associated with a reduced BP in both striatal regions putamen and caudate (Table 2). Conversely, we observed higher BP in the caudate of *COMT* Met/Met carriers. Since this association could have been driven by the interaction between COMT enzyme and levodopa, we verified that there were no differences between genotype groups in the LEDD at scan with ANOVA test (Val: 278 mg/day vs. Met/Met: 331 mg/day; *P* = 0.45). We also compared LEDD across genotypes for the other genetic factors and no differences were found. Lastly, we observed a trend for reduced caudate BP for H2/H2 carriers (*P* = 0.06). No relationship was found between DAT availability and *APOE* ε4 allele or benign variants in *GBA*.

**Table 1:** Descriptive values distributed by genotype for age of onset and percentage of putamen and caudate age-expected [^123^I]FP-CIT binding potential.

	n	AoO	Caudate	Putamen
**APOE**				
ε2+	32	55 ± 13	0.48 ± 0.29	0.30 ± 0.16
ε4+	60	54 ± 12	0.55 ± 0.25	0.35 ± 0.17
(ε2-,ε4-)	200	55 ± 13	0.61 ± 0.30	0.40 ± 0.19
**MAPT**				
H1/H1	166	54 ± 13	0.60 ± 0.31	0.39 ± 0.20
H1/H2	108	55 ± 14	0.58 ± 0.26	0.38 ± 0.16
H2/H2	20	56 ± 10	0.46 ± 0.29	0.33 ± 0.14
**COMT**				
Met/Met	52	57 ± 12	0.67 ± 0.30	0.43 ± 0.21
Val/Met	146	55 ± 14	0.57 ± 0.29	0.37 ± 0.17
Val/Val	98	53 ± 13	0.57 ± 0.29	0.37 ± 0.18
**SNCA**				
G/G	56	55 ± 14	0.53 ± 0.29	0.35 ± 0.17
A/G	149	55 ± 12	0.57 ± 0.29	0.38 ± 0.19
A/A	88	54 ± 14	0.64 ± 0.29	0.41 ± 0.17
**GBA**				
deleterious	31	50 ± 8	0.53 ± 0.31	0.33 ± 0.15
benign	17	52 + 11	0.58 ± 0.31	0.37 ± 0.21
non-carriers	250	55 ± 13	0.59 ± 0.29	0.38 ± 0.18

AoO: Age of disease onset

**Table 2:** Results for linear regressions of SPECT variables corrected for sex, age and disease duration.

	Caudate		Putamen	
	β (95% CI)	p	β (95% CI)	p
**APOE**				
ε2+ vs (ε2-,ε4-)	-0.13 (-0.22,-0.02)	**0.01**	-0.18 (-0.25,-0.05)	**0.002**
ε4+ vs (ε2-,ε4-)	-0.07 (-0.13,0.03)	0.20	-0.09 (-0.14, 0.007)	0.08
**MAPT**				
H1/H1 vs H2	-0.007 (-0.07,0.05)	0.83	-0.001 (-0.04,0.04)	0.94
H2/H2 vs H1	-0.11 (-0.23,0.004)	0.06	-0.04 (-0.11,0.04)	0.31
**COMT**				
Met/Met vs Val	0.09 (0.01,0.16)	**0.03**	0.04 (-0.004,0.09)	0.07
**SNCA**				
G vs A	-0.06 (-0.10,-0.02)	**0.006**	-0.03 (-0.06,-0.005)	**0.02**
**GBA**				
deleterious vs non-carriers	-0.14 (-0.24,-0.03)	**0.01**	-0.18 (-0.26,-0.07)	**0.001**
benign vs non-carriers	-0.02 (-0.16,0.11)	0.70	-0.03 (-0.17,0.09)	0.55

β: regression coefficient; CI: confidence interval

### Dementia

**Fig 1:**
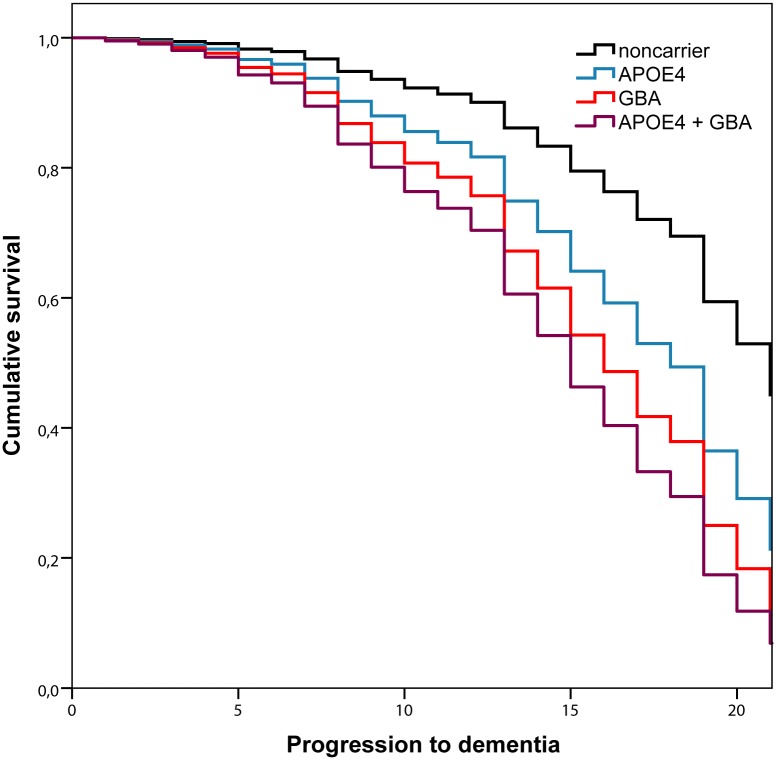
Survival plot of dementia onset. Lines represent the cumulative dementia-free survival in years from disease onset. GBA labels refer to patients carrying deleterious variants.

Of the 298 patients, 59 progressed to dementia after a mean average of 10 years from disease onset. Of those, 34 met the criteria for probable dementia and 25 met those for possible dementia. The cumulated probability of dementia was 25.7%. Cox regression analyses are presented in Table 3. We found that the development of dementia was influenced by the *APOE* ε4 allele (HR = 1.90; *P* = 0.03) and *GBA* deleterious variants (HR = 2.59; *P* = 0.01). The hazard ratio for the patients carrying both *APOE* ε4 and *GBA* deleterious variants was even higher although it did not reach significance due to the small number of cases (n = 6, HR = 2.95, *P* = 0.10). The survival curves for these two genetic factors and their combination are presented in Fig 1. Also, a trend for a protective effect was observed for *COMT* Met/Met (HR = 0.46; *P* = 0.07). Finally, no association was found for the *APOE* ε2 allele, *MAPT* H1/H1 genotype, *SNCA* polymorphism or *GBA* benign variants.

**Table 3:** Results for Cox regressions for the development of dementia corrected for sex and age of onset.

	HR (95% CI)	p
**APOE**		
ε4+	1.90 (1.05,3.44)	**0.03**
ε2+	1.19 (0.54,2.64)	0.67
(ε4-,ε2-)	Ref.	
**MAPT**		
H1/H1	0.83 (0.51,1.45)	0.48
H2	Ref.	
**COMT**		
Met/Met	0.46 (0.21,1.13)	0.07
Val	Ref.	
**SNCA**		
G/G	0.73 (0.35,1.59)	0.41
A	Ref.	
**GBA**		
deleterious	2.59 (1.16,5.76)	**0.01**
benign	1.69 (0.59,4.80)	0.32
non-carriers	Ref.	
**APOE + GBA**		
ε4+ and deleterious	2.95 (0.80,10.90)	0.10
non-carriers	Ref.	

HR: Hazards ratio, CI: Confidence Interval.

## Discussion

In this study, we found that striatal DAT availability levels in PD were influenced by *APOE* ε2 allele, *COMT* Val158Met, *SNCA* rs356219 and deleterious variants in *GBA*, whereas the development of dementia was influenced by the *APOE* ε4 allele and also by deleterious variants in *GBA*. Our results therefore suggest that *APOE*2, *COMT* and *SNCA* may be related to dopaminergic degeneration, while *APOE*4 may be related to other, non-dopaminergic degeneration mechanisms, and *GBA* may be implicated in both. Our findings support the dichotomy of the *dual-syndrome* hypothesis and provide new insights into the dissociation of the genetic factors which contribute to cognitive decline in PD.

The role of *APOE*2 in PD is controversial, some studies found a higher ratio of ε2 alleles in PD patients than in controls, although other studies do not share this finding [22, 24]. Similarly, the 'G' allele in *SNCA* polymorphism has been found overrepresented in PD [25]. There are no previous studies investigating the relationship between these genetic factors with striatal DAT, but our results suggest that both *APOE*2 and *SNCA* could have a negative effect on the dopaminergic pathway. We also observed a trend for reduced caudate BP fot H2/H2 carriers, although this trend should be further supported by other data sets since, to the best of our knowledge, no prior data on this relationship have been reported. Hence, our results suggest that *APOE2 and SNCA* may be implicated in PD pathogenesis and lead to a faster frontoexecutive impairment. No association with dementia onset was found, which is consistent with previuos data [11], and indicate that these *loci* do not play any role in the posterior cortical syndrome. We also found increased levels of caudate DAT in Met/Met carriers of *COMT* polymorphism. This is consistent with a ^18^F-DOPA PET study, which found higher presynaptic dopamine levels in frontal regions in Met/Met [26], and in controversy with a recent study that found higher levels of striatal FP-CIT BP in Val/Val [27]. However, this last result could be a false positive due to a small sample size (40 subjects in total, and only 3 Met/Met carriers).

We found *APOE*4 to be associated with a faster progression to dementia, but no such relationship was found for *MAPT* H1/H1. The observed effect size for *APOE* ε4 (HR = 1.90) was modest in comparison to that seen in AD but consistent with an existing meta-analysis in PD, which also suggests this allele has a moderate effect on PD dementia (OR = 1.74; 95% CI 1.36–2.23) [8]. Consistent with our data, Mata and colleagues' recent study noted the detrimental effect of *APOE* ε4 on cognition in PD, and no effect for *MAPT* H1/H1 [9]. Also, a previous study of PD in Spain discarded a relationship between *MAPT* H1/H1 and dementia [28]. On the other hand, a recent 10-year follow-up for the CamPaIGN cohort found a link between *MAPT* H1/H1 and dementia, and no link for *APOE* ε4 [11]. However, this discrepancy concerning *APOE* ε4 could arise from a lack of power, since only 38 demented PD patients and 35 non-demented PD patients were evaluated, and ε4 frequency was higher in the case of the demented (37% vs. 26%), although it did not reach a significant level.

Interestingly, we found that deleterious variants in *GBA* were associated to both reduced striatal BP and faster progression to dementia, possibly indicating that these variants play a role in both dopaminergic and non-dopaminergic degeneration processes. There are very few studies on dopaminergic imaging for PD *GBA* carriers, and these are limited to only a few cases;[29, 30] as of yet, no solid conclusions have therefore been drawn on the relationship between *GBA* and the dopaminergic system. Consistent with our observations, a recent study found a reduced glucocerebrosidase enzymatic activity in the substantia nigra of *GBA* carriers [31]. Clinical studies also support our results, having observed greater motor and cognitive impairment in PD patients with deleterious *GBA* variants (e.g. L444P, N370S), including a higher risk of progressing to Hoehn and Yahr stage 3 and dementia [15, 32]. Moreover, a recent study found executive and visuospatial deficits in these carriers, supporting our view that *GBA* might have a double-edged role in both dopaminergic and non-dopaminergic degeneration [33]. Also importantly, despite our bioinformatic analyses classified the variant E326K as benign, there are recent data suggesting the deleterious effect of E326K variant, including lower glucocerebrocidase activity [34] and worse performance in executive and visuospatial tasks in these carriers [33]. However, our data do not support the negative role of this variant. There were 5 patients heterozygous for E326K and 4 patients with compound heterozygosis with other deleterious variants, and none of them had reduced DAT binding in comparison with analogous non-carriers nor displayed signs of dementia after a mean follow-up of 14 years. We acknowledge that our sample size is limited to make conclusions about this variant but our observations on these 9 patients do not indicate that this variant should be classified as deleterious. Lastly, we also observed that the risk of *GBA* deleterious variants carriers to develop dementia was increased in combination with *APOE4* allele. However, this is just an observation and should be interpreted with caution since we only had 6 patients having both risk genotypes (3 of them got demented, and two of them were L444P carriers). Indeed, the result is not significant due to the lack of power.

In summary, *APOE*2, *COMT* Met, 'G' allele at *SNCA* rs356219 and deleterious variants in *GBA* contribute to dopaminergic degeneration in PD. These *loci* may therefore contribute to frontostriatal dysfunction. *APOE*4 and variants in *GBA* contribute to the development of dementia, and are possibly related to other non-dopaminergic processes. Different genetic risk genotypes produce different outcomes of the dual syndromes of cognitive impariment in PD, and deleterious variants in *GBA* may play a double-edged role in both. We acknowledge that the lack of exhaustive clinical and neuropsychological assessments for dementia is a potential limitation in our study. However, this population-based study was designed to overcome limitations of sample size in genetic studies and provide reliable effect sizes. Futher research will be able to verify the findings of this discovery sample, and will allow for more convincing conclusions.

## Supporting information

S1 Table List. (DOC)
