# Supplementary material for: Genetic factors influencing frontostriatal dysfunction and the development of dementia in Parkinson's disease
Source: PLoS One. 2017 Apr 11;12(4):e0175560. doi: 10.1371/journal.pone.0175560 (PMC5388496; doi:10.1371/journal.pone.0175560)
Supplement: S1 Table — (DOC) [file pone.0175560.s001.doc]

SUPPORTING INFORMATION

S1 Table. List of *GBA* variants grouped by potential pathogenicity according to *in-silico* analyses

|  | Allele | cDNA | Protein | Exon | n |
| --- | --- | --- | --- | --- | --- |
| deleterious | G195W | c.700G/T | p.Gly234Trp | 7 | 5 |
|  | S271G | c.928A/G | p.Ser310Gly | 8 | 2 |
|  | R262C / rs374117599 | c.901C>T | p.Arg301Cys | 8 | 1 |
|  | T369T / rs138498426 | c.1224G/ | p.Thr408Thr | 9 | 1 |
|  | W312R | c.1051T>A | p.Trp351Arg | 9 | 5 |
|  | N370S | c.1223A/G | p.Asn409Ser | 10 | 5 |
|  | D409H | c.1342G/C | p.Asp448His | 10 | 1 |
|  | -- | c.1264_1319del55 | p.Leu422fsx3 | 10 | 1 |
|  | L444P | c.1448T/C | p.Leu483Pro | 11 | 10 |
|  | V457D | c.1487T>A | p.Val496Asp | 11 | 2 |
|  | R496H | c.1604G>A | p.Arg535His | 12 | 2 |
|  | Total | | | | 35 |
| benign | -- | c.116-8C/T | -- | 4 | 4 |
|  | -- | c.588+7A>C | -- | 6 | 1 |
|  | I119I / rs14741115 | c.474C/T | p.Ile158Ile | 6 | 1 |
|  | L268L | c.921C/T | p.Leu307Leu | 8 | 2 |
|  | R262H / rs140955685 | c.902G/A | p.Arg301His | 8 | 1 |
|  | T369M / rs75548401 | c.1223C/T | p.Thr408Met | 9 | 3 |
|  | E326K / rs2230288 | c.1093G/A | p.Glu365Lys | 9 | 9 |
|  | -- | c.1388+10T>G | -- | 10 | 1 |
|  | T410T | c.1347G/C | p.Thr449Thr | 10 | 1 |
|  | A446T | c.1453G>A | p.Ala485Thr | 11 | 1 |
|  | A446A / rs199928507 | c.1455A>G | p.Ala485Ala | 11 | 1 |
|  | L449L | c.1464G/C | p.Leu488Leu | 11 | 2 |
|  | Total | | | | 27 |
